# Effect of Exercise Training on Prognosis in Community-acquired Pneumonia: A Randomized Controlled Trial

**DOI:** 10.1093/cid/ciae147

**Published:** 2024-03-18

**Authors:** Camilla Koch Ryrsø, Daniel Faurholt-Jepsen, Christian Ritz, Maria Hein Hegelund, Arnold Matovu Dungu, Bente Klarlund Pedersen, Rikke Krogh-Madsen, Birgitte Lindegaard

**Affiliations:** Department of Pulmonary and Infectious Diseases, Copenhagen University Hospital – North Zealand, Hillerød, Denmark; Centre for Physical Activity Research, Copenhagen University Hospital – Rigshospitalet, Copenhagen, Denmark; Department of Infectious Diseases, Copenhagen University Hospital, Rigshospitalet, Copenhagen, Denmark; Department of Clinical Medicine, University of Copenhagen, Copenhagen, Denmark; National Institute of Public Health, University of Southern Denmark, Copenhagen, Denmark; Department of Pulmonary and Infectious Diseases, Copenhagen University Hospital – North Zealand, Hillerød, Denmark; Department of Pulmonary and Infectious Diseases, Copenhagen University Hospital – North Zealand, Hillerød, Denmark; Centre for Physical Activity Research, Copenhagen University Hospital – Rigshospitalet, Copenhagen, Denmark; Centre for Physical Activity Research, Copenhagen University Hospital – Rigshospitalet, Copenhagen, Denmark; Department of Clinical Medicine, University of Copenhagen, Copenhagen, Denmark; Department of Infectious Diseases, Copenhagen University Hospital, Copenhagen, Denmark; Department of Pulmonary and Infectious Diseases, Copenhagen University Hospital – North Zealand, Hillerød, Denmark; Centre for Physical Activity Research, Copenhagen University Hospital – Rigshospitalet, Copenhagen, Denmark; Department of Clinical Medicine, University of Copenhagen, Copenhagen, Denmark

**Keywords:** community-acquired pneumonia, exercise training, admission, length of stay, readmission

## Abstract

**Objective:**

To investigate the effect of standard care (SoC) combined with supervised in-bed cycling (Bed-Cycle) or booklet exercises (Book-Exe) versus SoC in community-acquired pneumonia (CAP).

**Methods:**

In this randomized controlled trial, 186 patients with CAP were assigned to SoC (n = 62), Bed-Cycle (n = 61), or Book-Exe (n = 63). Primary outcome length of stay (LOS) was analyzed with analysis of covariance. Secondary outcomes, 90-day readmission, and 180-day mortality were analyzed with Cox proportional hazard regression and readmission days with negative-binominal regression.

**Results:**

LOS was −2% (95% CI: −24 to 25) and −1% (95% CI: −22 to 27) for Bed-Cycle and Book-Exe, compared with SoC. Ninety-day readmission was 35.6% for SoC, 27.6% for Bed-Cycle, and 21.3% for Book-Exe. Adjusted hazard ratio (aHR) for 90-day readmission was 0.63 (95% CI: .33–1.21) and 0.54 (95% CI: .27–1.08) for Bed-Cycle and Book-Exe compared with SoC. aHR for 90-day readmission for combined exercise was 0.59 (95% CI: .33–1.03) compared with SoC. aHR for 180-day mortality was 0.84 (95% CI: .27–2.60) and 0.82 (95% CI: .26–2.55) for Bed-Cycle and Book-Exe compared with SoC. Number of readmission days was 226 for SoC, 161 for Bed-Cycle, and 179 for Book-Exe. Incidence rate ratio for readmission days was 0.73 (95% CI: .48–1.10) and 0.77 (95% CI: .51–1.15) for Bed-Cycle and Book-Exe compared with SoC.

**Conclusions:**

Although supervised exercise training during admission with CAP did not reduce LOS or mortality, this trial suggests its potential to reduce readmission risk and number of readmission days.

**Clinical Trials Registration:**

NCT04094636.

## Teaser

Supervised exercise training during admission may not further reduce the already short admission length in patients with community-acquired pneumonia. However, exercise training during admission might have the potential to reduce the risk of readmission and the number of readmission days.

Community-acquired pneumonia (CAP) is a leading cause of admission from infectious causes worldwide [1, 2]. CAP is a significant burden to patients and healthcare systems, with 24% of patients being readmitted within 30 days [3]. Despite increased focus on mobilization, CAP is associated with excessive bed rest because patients spend more than 96% of their time in sedentary behavior, with few steps per day during admission [4–6]. Physical inactivity during admission with CAP has been associated with prolonged length of stay (LOS) and increased in-hospital and 30-day mortality risks [4]. Hospital-associated deconditioning is a severe concern, as up to 40% of patients with CAP lose muscle strength during admission [3], which increases the risk of functional decline and adverse outcomes [7–9].

Early progressive mobilization within 24 hours of admission in patients with CAP has been shown to reduce LOS without affecting readmission and mortality risks [10, 11]. Exercise training initiated during admission in patients with CAP, including endurance and resistance, has been shown to counteract the negative consequences of physical inactivity on muscle strength and functional status [12]. However, evidence on the effect of exercise training during admission on prognosis in patients with CAP is sparse.

We hypothesized that supervised in-bed cycling or exercise training according to a booklet (booklet exercise) [13] was superior to standard care in improving prognosis in patients with CAP. We aimed to investigate the effect of standard care combined with supervised in-bed cycling or booklet exercise compared with standard care in patients admitted with CAP on LOS, 90-day readmission, and 180-day mortality risks.

## METHODS

### Trial Design

This single-center, randomized, parallel-group, 3-arm, open-label, controlled trial (Clinicaltrials.gov: NCT04094636) was conducted at Copenhagen University Hospital – North Zealand, Denmark. The trial was approved by the scientific ethical committee at the Capital Region of Denmark (H-18024256). The study followed the Declaration of Helsinki and CONSORT guidelines [14].

### Patients

Clinicians or nurses screened patients through medical files within 24 hours of admission. Patients ≥18 years old with suspected CAP were eligible for enrollment. CAP was defined as a new infiltrate on chest x-ray or computed tomography scan together with a minimum of 1 symptom of CAP (ie, cough, chest pain, fever [≥38.0°C], hypothermia [<35.0°C], or dyspnea) [15]. Exclusion criteria were admission within the past 14 days, paralysis or inability to move the legs preventing exercise, expected LOS of ≤72 hours, immunosuppression (eg, ≥20 mg prednisolone-equivalent/day ≥14 days or other immunosuppressive drugs, former transplant, human immunodeficiency virus, chemotherapy ≤28 days), or terminal illness. On 11 March 2021, the inclusion criteria were broadened to include patients with CAP caused by severe acute respiratory syndrome coronavirus 2 (SARS-CoV-2). Patients provided written informed consent.

### Randomization and Blinding

Patients were randomly assigned (1:1:1) to standard care, standard care combined with in-bed cycling, or standard care combined with booklet exercise [16]. Randomization was performed in randomly permuted blocks with block sizes of 3 using computer-generated numbers [16]. Patients were assigned with concealed allocation by a random number sequence after baseline assessment. A researcher without involvement in testing and training generated the allocation sequence. Patients, assessors, and investigators were aware of the assignment. Healthcare professionals (clinicians, nurses, and physiotherapists) were unblinded.

### Interventions

The reporting of the interventions follows the Template for Intervention Description and Replication (TIDieR) checklist [17]. All patients received standard care according to Danish guidelines [18, 19], including ward rounds, nursing assistance, and physiotherapy if needed. In addition, the in-bed cycling and booklet exercise groups received daily supervised exercise training. The intervention was programmed as 30 minutes of exercise/day, including weekends. An exercise scientist, nurse, or physiotherapy student supervised each session face-to-face. Oxygen saturation (SpO_2_) and heart rate (HR) were measured before and throughout each session with a pulse oximeter (PalmSAT 2500 Pulse Oximeter, NONIN Medical Inc., USA) to ensure safety. Perceived exertion was evaluated with the Borg scale (6–20 points) [20]. The maximum age-related HR was calculated as maximumHR=220–age [21]. No target HR was set for exercise. If a patient felt dyspnea or SpO_2_ dropped >3% below resting SpO_2_ [22–24], exercise was temporarily interrupted. Exercise was continued if SpO_2_ returned to resting SpO_2_ within 5 minutes. After each session, the patient was observed for 5 minutes to ensure the return of resting SpO_2_.

The booklet exercise intervention included 30 minutes of supervised exercise using exercises from the booklet “*Syg men Sund og Aktiv”* (“*Sick but Healthy and Active”*) [13]. The booklet combines simple body weight-bearing resistance and walking exercises to improve balance, strength, and endurance (Supplementary Figure 1). Resistance exercises were performed at the bedside and adapted to the patient's abilities. The exercise instructor gradually increased the exercise intensity. Resistance training consisted of 2–3 sets of 8–10 repetitions.

The in-bed cycling intervention included 25 minutes of supervised continuous bedside cycle ergometer exercise (Lemco, Denmark) and 5 minutes of booklet exercise. Based on SpO_2_, HR, and Borg, exercise intensity gradually increased throughout admission. Patients who reported Borg ≤10 had an increase in exercise intensity the following session. For booklet exercise, this included increasing exercise time, sets, and repetitions. For in-bed cycling, pace, load, and exercise time were increased. The exercise was initiated within 48 hours of admission and performed in patient rooms or the hallway (data supplement contains a detailed description of interventions). No modifications were made to the intervention during the study.

### Outcomes

The primary outcome was LOS. Secondary outcomes were 90-day readmission and 180-day mortality. An explorative outcome was total readmission days within 90 days after discharge.

### Data Collection

Information about demographic characteristics, comorbidities, disease severity, and clinical outcomes was collected from medical records. The confusion, urea level, respiratory rate, blood pressure, and age ≥65 years score [25] was used to risk-stratify patients into mild (score 0–1), moderate (score 2), or severe (score 3–5) CAP. The Charlson Comorbidity Index [26] was used to assess comorbidities and was categorized as 0, 1, or ≥2 comorbidities. Medical records were used to collect data on LOS (from admission to discharge), admission to the intensive care unit, readmission, mortality, and adverse events (eg, falls, pulmonary embolisms). Readmission and mortality data were collected up to 90 days and 180 days after discharge.

### Sample Size

Based on previous results [10], a sample of 70 patients per group (210 patients in total) would provide 80% power to detect a mean difference of 2 days (6.5 to 4.5 days) in LOS between standard care and in-bed cycling or booklet exercise with a standard deviation (SD) of 3.5 days, an estimated dropout of 15%, and an α-level of 0.025 (2 comparisons against standard care). The inclusion began on 2 April 2019 and was terminated on 1 March 2022 [16].

### Statistical Analysis

Baseline characteristics are summarized as means with SD or medians with interquartile range (IQR) for continuous variables and counts with percentages for categorical variables. LOS was assessed using the intention-to-treat principle. Readmission and mortality were examined using available-case analysis (patients discharged alive). Analysis of covariance was used to quantify differences in LOS and included age, sex, number of comorbidities, confusion, urea level, respiratory rate, blood pressure, and age ≥65 years, and body mass index as covariates. Model assumptions, including normality, were tested using residual and quantile–quantile plots. Because LOS had a right-skewed distribution, it was logarithm-transformed. Regression coefficients were back-transformed to provide ratios. Kaplan–Meier plots and Cox proportional hazards regression were used to explore differences in time to 90-day readmission and 180-day mortality. The analysis was univariate and multivariate, adjusted for age, sex, and number of comorbidities. As post hoc analyses, the exercise groups were combined. Sensitivity analyses were performed, excluding patients with SARS-CoV-2 infection and those with exercise-induced SpO_2_ desaturation >3%. Results were reported as HRs with 95% confidence interval (CI) and *P* value. Negative-binominal regression explored differences in the number of readmission days within 90 days. Results were reported as incidence rate ratios with 95% CI and *P* value. All comparisons were 2-sided, with a significance level of .05, except for the primary outcome, where Bonferroni adjustment was applied. Statistical analyses were performed with IBM SPSS Statistics v.25 [27].

## RESULTS

**Figure 1.**
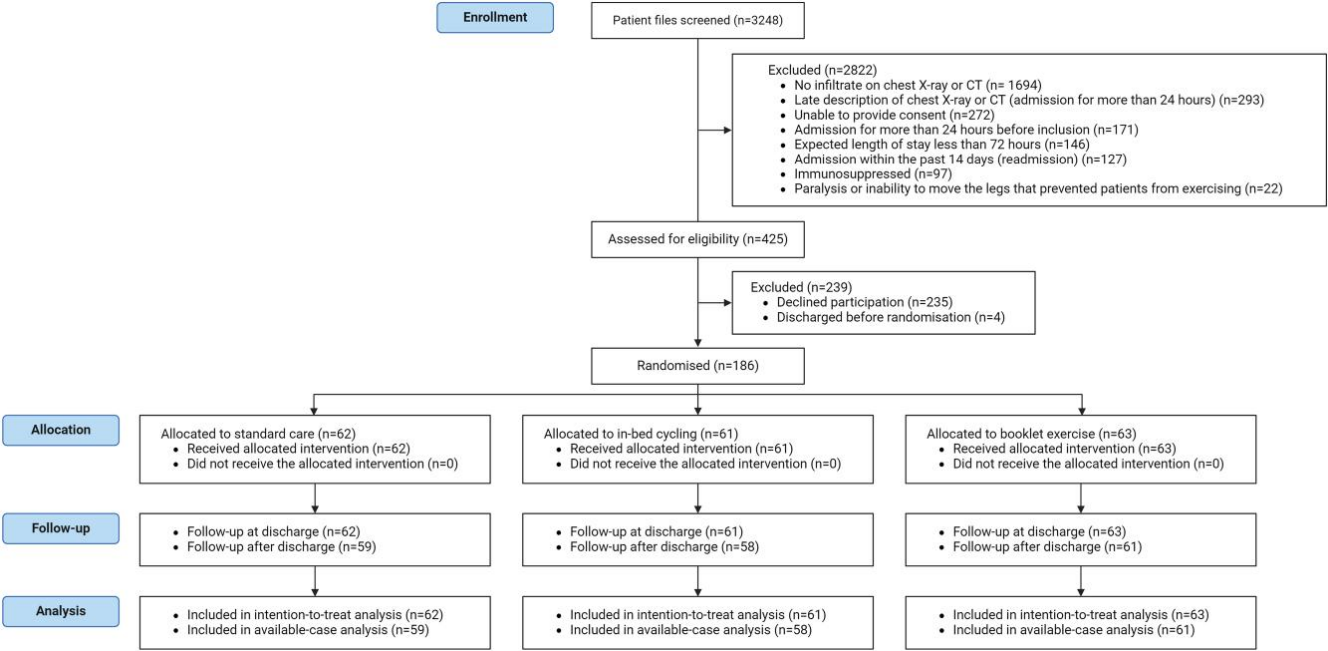
CONSORT flow diagram.

In total, 3248 patients were screened; 425 were approached for participation, of which 186 (43.8%) were enrolled in the study; 62 were assigned to standard care, 61 to in-bed cycling, and 63 to booklet exercise (Figure 1). Thus, 186 of the 210 planned patients were included without dropouts. The groups were balanced with respect to baseline characteristics (Table 1, Supplementary Table 1).

**Table 1.** Baseline Characteristics of 186 Patients Admitted With Community-acquired Pneumonia

	Standard Care (n = 62)	In-bed Cycling (n = 61)	Booklet Exercise (n = 63)
Age, mean ± SD, y	68 ± 13	70 ± 14	69 ± 14
Sex, male, No. (%)	30 (48)	30 (49)	23 (37)
Body mass index, mean ± SD kg/m^2^	26.9 ± 6.8	26.9 ± 6.0	28.1 ± 6.3
Charlson comorbidity index, median (IQR)	4 (2–5)	4 (2–5)	3 (2–5)
Comorbidities, No. (%)			
0	13 (21)	12 (20)	14 (22)
1	20 (32)	12 (20)	15 (24)
≥2	29 (47)	37 (61)	34 (54)
Chronic obstructive pulmonary disease	23 (37)	18 (30)	18 (29)
Other chronic respiratory diseases	14 (23)	16 (26)	18 (29)
Heart failure	6 (10)	12 (20)	11 (18)
Other chronic heart diseases	28 (45)	34 (56)	39 (62)
Diabetes	8 (13)	10 (16)	8 (13)
Cancer	7 (11)	9 (15)	4 (6)
Peptic ulcer disease	4 (7)	8 (13)	11 (18)
Cerebrovascular disease	4 (7)	4 (7)	9 (14)
Connective tissue disease	3 (5)	4 (7)	2 (3)
Peripheral vascular disease	3 (5)	3 (5)	2 (3)
Chronic kidney disease	1 (2)	1 (2)	2 (3)
Chronic liver disease	1 (2)	0 (0)	1 (2)
CURB-65, No. (%)			
0–1	36 (58)	35 (57)	36 (57)
2	21 (34)	21 (34)	22 (35)
3–5	5 (8)	5 (8)	5 (8)

Abbreviation: CURB-65, confusion, urea level, respiratory rate, blood pressure, and age ≥65 y [17]; IQR, interquartile range.

### Exercise Training

Across 508 sessions, 124 patients exercised for 141 hours and 15 minutes. In the in-bed cycling and booklet exercise groups, 72.1% and 74.6% of patients agreed to every session, and 92.4% and 92.7% of sessions were shortened. The mean exercise duration was 16 ± 10 and 15 ± 9 minutes/day for the in-bed cycling and booklet exercise groups (Table 2, Supplementary Figure 2). The mean distance the in-bed cycling group biked was 4.3 ± 2.9 km/day. Borg, HR, exercise intensity, SpO_2_, or average desaturation during exercise were similar between the groups. Exercise tolerance increased throughout admission. From a mean exercise duration of 14 ± 11 and 14 ± 10 minutes/day on day 1 to 20 ± 8 and 19 ± 9 minutes/day on day 3 in the in-bed cycling and booklet exercise groups (Supplementary Figure 2). No exercise-related adverse events occurred. In 20 of 508 exercise sessions (3.9%), SpO_2_ dropped >3% below resting SpO_2_. The study scored 10 on the TIDieR 12-item checklist (Supplementary data).

**Table 2.** Exercise Data From the in-bed Cycling and Booklet Exercise Groups

	In-bed Cycling (n = 61)	Exercise Booklet (n = 63)
Patients participating in every exercise session, No. (%)	44 (72)	47 (75)
Exercise time, mean ± SD, min/d	16 ± 10	15 ± 9
Number of shortened exercise sessions, No. (%)	255 (92)	215 (93)
Distance, mean ± SD, km/d	4.3 ± 2.9	–
Borg, mean ± SD, score	14 ± 1	13 ± 2
Heart rate, mean ± SD, beats/min	98 ± 14	97 ± 17
Percent of maximal heart rate, mean ± SD, %	66 ± 11	63 ± 9
Saturation, mean ± SD, %	92 ± 4	93 ± 4
Average desaturation, mean ± SD, %	−3 ± 3	−3 ± 2

Abbreviation: SD, standard deviation.

### LOS, Readmission, and Mortality

LOS was 6 days (IQR 4–9) in standard care compared with 5 (IQR 4–8) and 6 days (IQR 3–9) in the in-bed cycling and booklet exercise groups (Table 3). Compared with standard care, LOS was −2% (95% CI: −24 to 25) and −1% (95% CI: −22 to 27, Table 4) for the in-bed cycling and booklet exercise groups. At discharge, 6 patients (10%) were referred to postdischarge rehabilitation in the standard care group compared with 2 (3%) and 5 (8%) patients in the in-bed cycling and booklet exercise groups (Table 2).

**Table 3.** Clinical Outcome and Prognosis in Patients Admitted With Community-acquired Pneumonia

	Standard Care (n = 62)	In-bed Cycling (n = 61)	Booklet Exercise (n = 63)
Length of stay, median (IQR), d	6 (4–9)	5 (4–8)	6 (3–9)
Oxygen therapy, No. (%)	38 (61)	36 (59)	41 (65)
High-flow therapy, No. (%)	8 (13)	8 (13)	5 (8)
Noninvasive ventilation, No. (%)	2 (3)	0 (0)	4 (6)
Intensive care unit, No. (%)	2 (3)	2 (3)	1 (2)
Postdischarge rehabilitation, No. (%)	6 (10)	2 (3)	5 (8)
Mortality, No. (%)			
In-hospital mortality	3 (5)	3 (5)	2 (3)
30-d mortality	2 (3)	2 (3)	1 (2)
90-d mortality	4 (7)	3 (5)	6 (10)
180-d mortality	6 (10)	6 (10)	6 (10)
Readmission, No. (%)			
30-d readmission	16 (27)	8 (14)	11 (18)
90-d readmission	21 (36)	16 (28)	13 (21)
Total readmissions, No.	41	31	23
Readmission count, No. (%)			
1	14 (24)	6 (10)	5 (8)
2	3 (5)	5 (9)	6 (10)
3	0 (0)	5 (9)	2 (3)
≥4	4 (7)	0 (0)	0 (0)
Total readmission days	226	161	179
Readmission duration, No. (%)			
≤10 d	14 (67)	11 (69)	7 (54)
11–20 d	2 (10)	3 (19)	2 (15)
21–30 d	3 (14)	1 (6)	3 (23)
>30 d	2 (10)	1 (6)	1 (8)
Cause of readmission, No. (%)			
Pulmonary	10 (48)	12 (75)	6 (46)
Cardiovascular	4 (19)	2 (13)	1 (8)
Neurological	2 (10)	1 (6)	1 (8)
Malignant	1 (5)	0 (0)	2 (15)
Other	4 (19)	1 (6)	3 (23)

Abbreviation: IQR, interquartile range.

**Table 4.** Percentage Decrease in Length of Stay and Risk of 90-day Readmission and 180-day Mortality for the in-bed Cycling, Booklet Exercise, and Combined Exercise Training Groups, Compared With Standard Care

	Model 1		Model 2		Model 3		Model 4	
	Parameter Estimates (95% CI)	*P* value	Parameter Estimates (95% CI)	*P* value	Parameter Estimates (95% CI)	*P* value	Parameter Estimates (95% CI)	*P* value
***Intention-to-treat analyses***								
*Length of stay*								
Standard care, n = 62	Ref.		Ref.		Ref.		Ref.	
In-bed cycling, n = 61	0.98 (.76–1.26)	.85	0.96 (.75–1.23)	.74	0.97 (.76–1.24)	.81	0.98 (.76–1.25)	.85
Booklet exercise, n = 63	0.99 (.77–1.28)	.96	1.00 (.78–1.27)	.99	1.00 (.79–1.28)	.98	0.99 (.78–1.27)	.94
								
Standard care, n = 62	Ref.		Ref.		Ref.		Ref.	
Exercise, n = 124	0.98 (.79–1.23)	.89	0.98 (.79–1.21)	.84	0.99 (.80–1.22)	.90	0.98 (.80–1.22)	.88

	Model 1		Model 2		Model 3		Model 4	
	Hazard Ratio (95% CI)	*P*-value	Hazard Ratio (95% CI)	*P*-value	Hazard Ratio (95% CI)	*P*-value	Hazard Ratio (95% CI)	*P*-value
***Available-case analyses***								
*90-d readmission*								
Standard care, n = 59	Ref.		Ref.		Ref.			
In-bed cycling, n = 58	0.70 (.37–1.35)	.29	0.65 (.34–1.25)	.20	0.63 (.33–1.21)	.17		
Booklet exercise, n = 61	0.54 (.27–1.08)	.08	0.55 (.27–1.10)	.09	0.54 (.27–1.08)	.08		
								
Standard care, n = 59	Ref.		Ref.		Ref.			
Exercise, n = 119	0.62 (.35–1.09)	.10	0.60 (.34–1.05)	.08	0.59 (.33–1.03)	.06		
*180-d mortality*								
Standard care, n = 59	Ref.		Ref.		Ref.			
In-bed cycling, n = 58	1.01 (.33–3.14)	.98	0.88 (.28–2.75)	.83	0.82 (.26–2.55)	.73		
Booklet exercise, n = 61	0.98 (.32–3.05)	.98	0.93 (.30–2.89)	.90	0.84 (.27–2.60)	.76		
								
Standard care, n = 59	Ref.		Ref.		Ref.			
Exercise, n = 119	1.00 (.37–2.66)	>.99	0.91 (.34–2.42)	.84	0.83 (.31–2.21)	.71		

Abbreviation: 95% CI, 95% confidence interval.

The outcome variable length of stay was logarithm-transformed before analysis, and the regression coefficients were back-transformed to provide ratios. Data were analyzed with an analysis of covariance to quantify differences in length of stay between the standard care group and the in-bed cycling or booklet exercise group. The outcome variables readmission and mortality were analyzed with Cox proportional hazard regression to assess differences in time to readmission and mortality between the standard care group and the in-bed cycling and booklet exercise groups. Additional analyses were made with a combined exercise training group, which is the in-bed cycling and booklet exercise groups combined as 1 group. Model adjustments according to the different models: model 1: unadjusted model; model 2: adjusted for age and sex; model 3: adjusted for age, sex, and number of comorbidities (0, 1, or ≥2); and model 4: adjusted for age, sex, CURB-65 score, body mass index, and number of comorbidities (0, 1, or ≥2).

**Figure 2.**
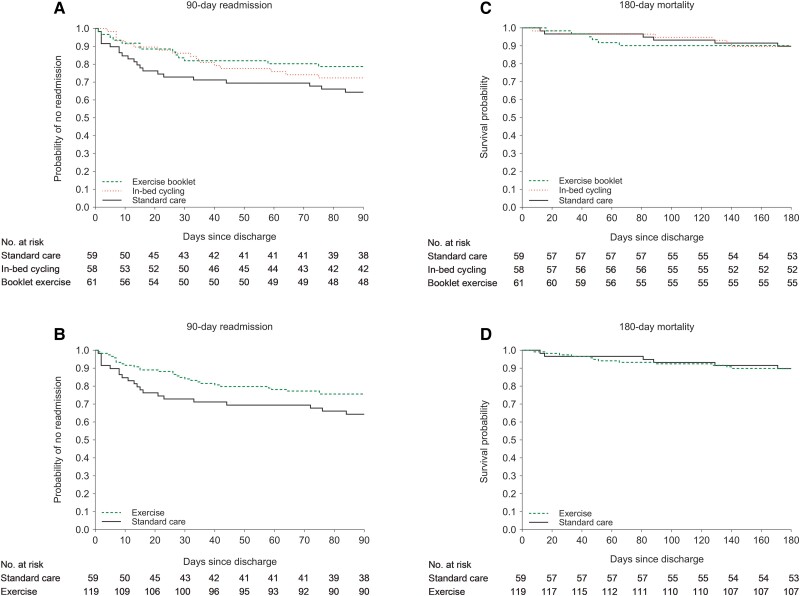
Kaplan–Meier estimation of 90-d readmission and 180-d mortality. Note: Kaplan–Meier estimation of 90-d readmission (*A*) and 180-d mortality (*C*) for standard care (black solid line), in-bed cycling (red dashed line), and booklet exercise (green dotted line) groups. Kaplan–Meier estimation of 90-d readmission (*B*) and 180-d mortality (*D*) for standard care (black solid line) and the combined exercise training (green dotted line) group.

The 90-day readmission rate was 35.6% in standard care compared with 27.6% and 21.3% in the in-bed cycling and booklet exercise groups (Table 3, Figure 2*A*). Pulmonary conditions (eg, CAP, coronavirus disease 2019, and acute exacerbations of chronic obstructive pulmonary disease) caused 56% of the readmissions. The adjusted hazard ratio (aHR) for 90-day readmission was 0.63 (95% CI: .33–1.21) in the in-bed cycling and 0.54 (95% CI: .27–1.08; Table 4) in the booklet exercise groups, compared with standard care. Post hoc analysis with the combined exercise group resulted in aHR for 90-day readmission of 0.59 (95% CI: .33–1.03; Table 4, Figure 2*B*) compared with standard care. In a sensitivity analysis of patients with non-SARS-CoV-2 CAP, aHR for 90-day readmission was 0.65 (95% CI: .36–1.19; Supplementary Table 2) for the combined exercise group compared with standard care. Patients who maintained an acceptable SpO_2_ during exercise had an aHR of 0.59 (95% CI: .33–1.05; Supplementary Table 3) compared with standard care. Standard care had 226 readmission days within 90 days, whereas in-bed cycling and booklet exercise had 161 and 179 days, respectively (ie, 47 and 65 fewer days in the booklet exercise and in-bed cycling groups than standard care) (Table 3). The incidence rate ratio for total readmission days was 0.73 (95% CI: .48–1.10) and 0.77 (95% CI: .51–1.15) in the in-bed cycling and booklet exercise groups compared with standard care.

The 180-day mortality rate was 10.2% in standard care compared with 10.3% and 9.8% in the in-bed cycling and booklet exercise groups (Table 3, Figure 2*C*). The aHR for 180-day mortality was 0.84 (95% CI: .27–2.60) and 0.82 (95% CI: .26–2.55, Table 4) for the in-bed cycling and booklet exercise groups compared with standard care.

## DISCUSSION

Among patients admitted with CAP, supervised in-bed cycling and booklet exercise had no effect on LOS. Similarly, exercise training had no statistically significant effect on 90-day readmission risk; however, in-bed cycling and booklet exercise may be associated with fewer readmission days within 90 days after discharge compared to standard care. The 180-day mortality risk was similar between standard care and the exercise groups.

Recently, early discharge initiatives have gained attention [28]. However, LOS was unaffected by exercise training in our study. In the design, exercise training was expected to reduce LOS by 2 days, which was based on previous observations from patients with CAP (6.9 to 5.8 days) with early progressive mobilization [10]. Thus, we believed supervised exercise training would be more effective than mobilization in reducing LOS. However, a quality study was initiated in 2018 at Copenhagen University Hospital – North Zealand to improve the treatment of CAP, including early discharge [29]. Along with this, a study of patients with CAP showed that adherence to guidelines reduces LOS by 1.8 days (6.5 to 4.7 days) [30]. Thus, increased focus on guidelines may have improved treatment efficacy, leading to shorter admissions but high 30-day readmission rate [3, 4, 31, 32]. Likely, LOS had already been shortened as much as possible in patients with CAP, with a median LOS of 5 days [33], like our findings. A further reduction in LOS with exercise may be unrealistic.

Readmissions after CAP remain challenging, with 24% being readmitted within 30 days [3, 4, 31]. However, there was no sign of a statistically significant association between in-bed cycling or booklet exercise and 90-day readmission risk. Because no difference between the 2 exercise groups was found, they were combined in a post hoc analysis, demonstrating a nonsignificant association with 90-day readmission compared with standard care (*P* = .06). Though our study was not powered to show an effect of exercise on readmissions, the lower number of readmissions within 90 days after discharge with exercise training could be clinically relevant. Thus, the statistically nonsignificant association between exercise (in-bed cycling and booklet exercise) and 90-day readmission is most likely the result of the small sample size, as the post hoc analysis showed a tendency (*P* = .06) toward a reduced 90-day readmission risk with exercise training. The tendency toward a reduced 90-day readmission risk with exercise remained despite excluding patients who desaturated >3% during exercise in a sensitivity analysis (*P* = .07). These findings suggest that patients who exercised at moderate exercise intensity (ie, not pushed beyond the point of exhaustion) might be those who benefited most from exercise training during admission. However, exercise training may be associated with fewer readmission days within 90 days after discharge (ie, 47 and 65 days) compared with standard care. Unplanned readmissions are associated with poor health outcomes and increased healthcare costs [34, 35]. In patients with CAP, only 10% to 14% of unplanned 30-day readmissions are deemed preventable [34, 36]. However, exercise interventions might have immense potential to improve the prognosis of patients with CAP. In times of resource constraints, cost-effective healthcare initiatives are warranted. Healthcare systems are financially burdened by CAP, partly because of high readmission rates [3, 4, 31]. According to the Danish Health Data Authority, healthcare costs associated with CAP are approximately $6000 per admission [37]. Therefore, our finding that exercise training during admission with CAP was associated with a tendency toward a reduced readmission risk and fewer readmission days may have clinical and economic implications.

The mechanisms by which exercise training during admission might reduce the risk of readmission and the number of readmission days in patients with CAP are still unknown. However, in elderly patients, exercise during admission offsets the deleterious consequences of prolonged bed rest on muscle strength and functional status but does not affect readmission risk [38–40]. Further, we have shown that even modest increases in physical activity after discharge in patients with CAP were associated with reduced 30-day readmission risk [4]. Our findings suggest that exercise training might alleviate readmissions in patients with CAP. The safety of initiating exercise training on admission has been an area of interest. However, we found no differences in 180-day mortality between standard care and exercise training.

### Strengths and Limitations

As evidence on the effects of supervised exercise training during admission with CAP is sparse, novelty is the major strength of our study. In contrast to our study, most exercise interventions in the acute care setting involve older patients admitted with cardiovascular, gastrointestinal, or infectious diseases but without respiratory limitations [38, 39]. Because of the respiratory impact of CAP and the risk of exercise-induced desaturation, exercise intensity was adapted to the ventilatory limitation. For safety, we interrupted exercise if SpO_2_ dropped >3% below resting SpO_2_. To maintain a resting SpO_2_ of 94%–98% (88%–92% for patients with chronic obstructive pulmonary disease and CAP) [41], oxygen therapy is often needed in patients with CAP. Because pulse oximetry is accurate within 2%–3% of arterial blood gas values [42], a >3% drop in SpO_2_ was chosen as the cutoff for exercise termination [23, 24].

Our study also had some limitations. First, the study was terminated on 1 March 2022, as defined in the protocol [16], with 186 of 210 planned patients included, though protocol adjustments were made to meet the intended sample size. The inclusion criteria were expanded to SARS-CoV-2–related CAP; this was based on our previous study [43], and the recruitment was prolonged by 7 months. In our power and sample size calculation, the estimated effect of exercise was based on a LOS of 6.5 days for standard care (6.5 vs 4.5 days). Thus, with a shorter LOS of 6 days in the standard care group and 60 patients in each group, we only have 64% power to detect the expected 4.5-day LOS in exercise training groups (1.5-day shortening). Though a 1-day reduction would be clinically meaningful, it would require a considerably larger sample size. However, we acknowledge that the study was underpowered in terms of showing the effect of exercise training on LOS. A second limitation is the lack of blinding for the healthcare personnel and outcome assessors. Third, patients were scheduled for 30 minutes of daily exercise; however, both groups exercised for 15–16 minutes/day. No cutoff for exercise adherence was set; this may have decreased the effect of our interventions because of a lower exercise volume than with a prespecified cutoff. The lower exercise volume was likely due to some patients being too sick to exercise despite consent to participate. Delayed inclusion or initiation of exercise might have enhanced adherence to the prescribed exercise. However, early initiation of exercise was required due to the short LOS.

## CONCLUSION

Although supervised exercise training during admission with CAP may not further reduce the already short admission, this trial suggests its potential to reduce readmission risk and the number of readmission days without increasing adverse events.

## Supplementary Data

Supplementary materials are available at *Clinical Infectious Diseases* online. Consisting of data provided by the authors to benefit the reader, the posted materials are not copyedited and are the sole responsibility of the authors, so questions or comments should be addressed to the corresponding author.

## Supplementary Material

ciae147_Supplementary_Data

## Notes

***Author Contributions.*** C. K. R. and M. H. H. had full access to all the data and take responsibility for the integrity of the data and the accuracy of the analysis. Concept and design: C. K. R., D. F.-J., B. K. P., C. R., R. K.-M., and B. L. Acquisition, analysis, or interpretation of data: All authors. Drafting of the manuscript: C. K. R. Critical revision of the manuscript for important intellectual content: All authors. Statistical analysis: C. K. R. and C. R. Obtained funding: B. L., R. K.-M., C. K. R., and B. K. P. Administrative, technical, or material support: C. K. R., D. F.-J., B. K. P., C. R., M. H. H., A. M. D., R. K.-M., and B. L. Supervision: D. F.-J., B. K. P., C. R., R. K.-M., and B. L.

***Acknowledgments.*** Malene Pilegaard Schønnemann, Hanne Hallager, Christina Brix, and students in medicine, physiotherapy, and human physiology are acknowledged for their technical assistance.

***Data sharing statement.*** Relevant anonymized participant data will be made available for others on a reasonable request to the corresponding author. The lead and corresponding authors affirm that the manuscript is an honest, accurate, and transparent account of the study being reported; that no important aspects of the study have been omitted; and that any discrepancies from the study as planned and registered have been explained.

***Financial support*.** The Centre for Physical Activity Research is supported by TrygFonden (grant numbers: ID 101390, ID 20045, and ID 125132). The study was supported by grants from the Copenhagen University Hospital – North Zealand (grant number: none), Hillerød, Denmark, and the Grosserer L.F. Foghts Fond (grant number: none). The funders had no role in the design and conduct of the study; collection, management, analysis, and interpretation of the data; preparation, review, or approval of the manuscript; and decision to submit the manuscript for publication.
