# Supplementary material for: Effect of Exercise Training on Prognosis in Community-acquired Pneumonia: A Randomized Controlled Trial
Source: Clin Infect Dis. 2024 Mar 18;78(6):1718–26. doi: 10.1093/cid/ciae147 (PMC11175663; doi:10.1093/cid/ciae147)
Supplement: ciae147_Supplementary_Data [file ciae147_supplementary_data.docx]

**Data Online Supplementary Files**

**A detailed description of the interventions**

The Template for Intervention Description and Replication (TIDieR) Checklist [1] has been used to report the content of each intervention. No modifications were made to the intervention during the study.

***Standard care:*** Patients who were randomly assigned to the standard care group received usual care for patients admitted with CAP in Denmark, which included mobilisation (i.e., sitting out of bed for at least 20 minutes per day) and physiotherapy, which were prescribed by the attending physician when needed [2].

A physiotherapy session included:

- Evaluation of the CAP severity (i.e., secrete problems or dyspnea).
- Instructions on respiratory therapeutic techniques to improve respiration (i.e., secretion clearance techniques, management of dyspnea, positive expiratory pressure (PEP) therapy, continuous positive airway pressure (CPAP) therapy, etc.).
- Evaluation of the patient's own resources in relation to carrying out respiratory physiotherapy themselves.
- Evaluation of the patient's functional level (e.g., need for assistance to change positions, need for assistance for mobilisation out of bed, or use of a walking aid during mobilisation).
- Evaluation of the patient's own resources in relation to changing positions and being physically active during admission.
- Evaluation of the patient's need for physiotherapeutic rehabilitation during and/or after discharge [2].

Depending on the physiotherapeutic examination, patients will be treated accordingly [2].

- Well-mobilised patients, without any secret, will be informed about the importance of being physically active during admission.
- Well-mobilised patients with moderate-to-severe CAP and secret problems will be informed about the importance of being physically active and instructed in relevant respiratory physiotherapeutic techniques.
- For exhausted patients with moderate-to-severe CAP who suffer from severe breathlessness, the physiotherapist will offer coughing techniques, CPAP and/or PEP treatment, and assistance during mobilisation, physical activity, and rehabilitation.

***Exercise interventions:*** To ensure safety during exercise, resting oxygen saturation (SpO_2_) and heart rate measurements were performed before each session with a pulse oximeter (PalmSAT 2500 Pulse Oximeter, NONIN Medical Inc., USA) and monitored continuously during each session to ensure that patients were not pushed beyond the point of exhaustion.

The attending physician determined the acceptable SpO_2_, which was obtained from patient files. Patients were allowed to exercise with supplementary oxygen in order to maintain an acceptable SpO_2_ during exercise. Further, patients were allowed to decide if they wanted to stop exercising before reaching 30 minutes per day. Perceived exertion was evaluated after each session with the Borg rating of the perceived exertion scale [3]. The Borg scale is a 6 to 20 rating scale, with a score of 6 being “no exertion at all” and a score of 20 being “maximal exertion” [3,4]. Further, a score of 12 to 14 on the Borg scale is “somewhat hard,” and exercise is performed at a moderate intensity level [3,4]. The estimated maximum age-related heart rate was based on the equation: maximum heart rate = 220 – age. No target heart rate was set for the exercise training. An exercise session was temporarily interrupted if a patient felt dyspnea or if SpO_2_ dropped >3% below resting SpO_2_. If SpO_2_ returned to resting levels within 5 minutes of rest, the exercise session was continued. After each session, the patient was observed for 5 minutes to ensure a return to resting SpO_2_.

*Booklet exercise:* The booklet exercise intervention consisted of a multicomponent exercise training programme using exercises from the exercise booklet entitled *“Syg men Sund og Aktiv”* [*“Sick but Healthy and Active”* in English] [5] (Figure S1). The booklet exercise consisted of face-to-face, supervised progressive, functional resistance exercises and walking exercises for seven consecutive days, including weekends. An exercise scientist with experience in exercise training for patients with cardiopulmonary diseases was responsible for the exercise intervention. Each exercise session was supervised by either an exercise scientist, a study nurse, or physiotherapy students. The exercise scientist trained and oversaw the exercise staff (study nurses and physiotherapy students) to ensure intensity progression and proper delivery of the exercise intervention.

The booklet exercise intervention was designed as approximately 30 minutes of total exercise per day, with approximately 5–10 minutes of walking/endurance exercise and approximately 20–25 minutes of resistance exercise. The supervised multicomponent exercise training programme consisted of upper and lower-body resistance exercises tailored and personalised to the patient’s functional capacity. The resistance exercises were performed with body weight and without additional exercise equipment in the patient's room at the bedside, tailored to the patient’s functional level. The resistance exercises focused on the major upper and lower limb muscles. Each exercise session included 2 exercises for the lower limbs (e.g., squats, lunges, sit-to-stand exercises), 2 exercises for the upper limbs (e.g., push-ups, shoulder flexion, chest press), and 2 exercises for the core muscles (e.g., standing arm and leg extension, seated ab exercise, seated lower back extension) with 2–3 sets of 8–10 repetitions. The resistance exercises were combined with walking exercises for 5–10 minutes (e.g., walking on the spot, continuous walking in the hallway, or interval walking in the hallway, i.e., 1 minute of brisk walking followed by 1 minute of slow walking × 4 times, in total 8 minutes). The exercise instructor supervising the exercise session was responsible for progressively increasing the exercise intensity. To induce a continuous exercise stimulus, the increasing exercise intensity was based on SpO_2_ and heart rate during exercise and the Borg score after each session [3]. If a patient reported a Borg score ≤10, the exercise intensity increased the following exercise session. For the booklet exercise intervention, this included increasing the exercise time (up to 30 minutes), number of sets (up to 3 sets), and number of repetitions (up to 10 repetitions). However, care was taken to ensure that every exercise was executed correctly.

The booklet exercise intervention was chosen as early progressive mobilisation has been shown to reduce the length of stay in patients admitted with CAP [6]. Thus, we believed that booklet exercises that combine the principles of progressive mobilisation with more demanding activities (i.e., walking or body weight-barring resistance exercises) would be beneficial in improving the prognosis of patients with CAP. The booklet exercise intervention was initiated within 48 hours after admission.

*In-bed cycling:* The in-bed cycling intervention consisted of approximately 30 minutes of face-to-face supervised exercise training per day, with 25 minutes of continuous exercise on a bed bike (Lemco Rehab & Fysio, Helsingør, Denmark, Figure S1) and 5 minutes of booklet exercise, with typically 1–2 resistance exercises (1 exercise for the upper and lower limbs each) with 2–3 sets of 8–10 repetitions. Each exercise session was supervised by an exercise scientist, a study nurse, or a physiotherapy student and performed in the patient room, either in bed or at the bedside. An exercise scientist with experience in exercise training for patients with cardiopulmonary diseases was responsible for the exercise intervention. Each exercise session was supervised by either the exercise scientist, a study nurse, or physiotherapy students. The exercise scientist trained and oversaw the exercise staff (study nurses and physiotherapist students) to ensure intensity progression and proper delivery of the exercise intervention. Each exercise session was tailored and personalised to the patient's functional capacity. The exercise instructor supervising the exercise session was responsible for progressively increasing the exercise intensity. To induce a continuous exercise stimulus, the increasing exercise intensity was based on SpO_2_ and heart rate during exercise and the Borg score after each session [3]. If a patient reported a Borg score ≤10, the exercise intensity increased the following exercise session. For the in-bed cycling intervention, pace (rounds per minute), load (level 0–4), and exercise time were increased (up to 30 minutes). However, care was taken to ensure that every exercise was executed correctly.

The in-bed cycling intervention was chosen because it was thought to enhance early recovery of lower limb muscle strength. In severely ill patients admitted to the intensive care unit, in-bed cycling has been demonstrated to induce superior effects on muscle force and walking distance compared to standardised physiotherapy [7]. It was thought that in-bed cycling would allow the patients to get out of bed earlier and start walking around the department. The in-bed cycling intervention was initiated within 48 hours after admission.

***Safety measures:*** Patients with terminal illnesses (i.e., discontinuation of active treatment within the first 48 hours of admission) were excluded from the study. If a patient was suspected of having pulmonary thromboembolism, they were allowed to exercise once the suspicion of pulmonary thromboembolism was disproved by a computed tomography angiography scan. If pulmonary thromboembolism was confirmed, the patient should have been in treatment with heparin for at least 24 hours before we resumed the exercise training. Further, patients with severe dyspnea were not allowed to exercise during treatment with non-invasive ventilation. However, if the attending physician agreed, they were allowed to exercise between treatments with non-invasive ventilation.

**Figure S1.** Pictures of patients performing in-bed cycling and booklet exercises**.** In-bed cycling (A) is performed on a bed bike. Booklet exercises with body-weight barring resistance exercises are performed at the bedside and include upper and lower limb exercises. Examples of booklet exercises are push-ups (B), sit-to-stand (C), squats (D), and lunges (E).

**
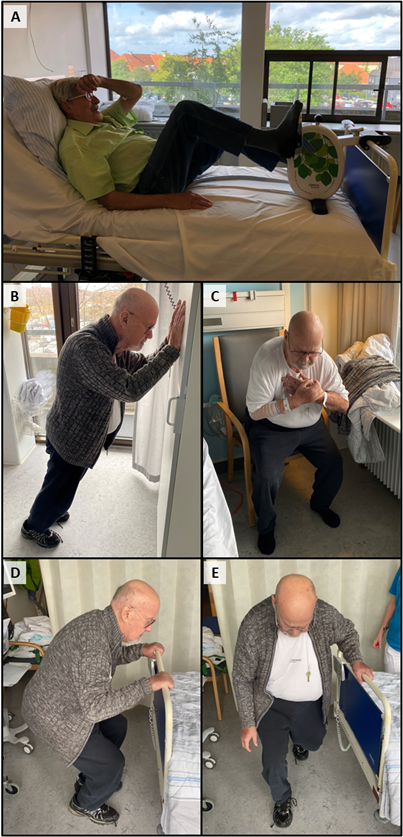
**

**Table S1.** Aetiology of CAP in the standard of care and the two exercise groups.

| The pathogen, No. (%) | Standard care (n = 62) | In-bed cycling (n = 61) | Booklet exercise (n = 63) |
| --- | --- | --- | --- |
| *Streptococcus pneumoniae* | 5 (8) | 7 (12) | 3 (5) |
| *Haemophilus influenzae* | 4 (7) | 4 (7) | 4 (6) |
| *Legionella pneumophila* | 1 (2) | 5 (8) | 2 (3) |
| *Staphylococcus aureus* | 2 (3) | 4 (7) | 0 (0) |
| *Staphylococcus hominis* | 0 (0) | 3 (5) | 2 (3) |
| *Escherichia coli* | 2 (3) | 1 (2) | 1 (2) |
| *Mycoplasma pneumoniae* | 2 (3) | 1 (2) | 0 (0) |
| *Moraxella catarrhalis* | 1 (2) | 2 (3) | 0 (0) |
| *Pseudomonas aeruginosa* | 0 (0) | 0 (0) | 2 (3) |
| SARS-CoV-2 | 8 (13) | 9 (15) | 9 (14) |
| Parainfluenza | 2 (3) | 0 (0) | 1 (2) |
| Respiratory syncytial virus | 1 (2) | 1 (2) | 1 (2) |
| Influenza virus A | 0 (0) | 1 (2) | 1 (2) |
| Human metapneumovirus | 0 (0) | 1 (2) | 1 (2) |
| Other | 14 (23) | 13 (21) | 10 (16) |

**Figure S2.** Mean exercise time per session in the two exercise groups.

**A**

**B**

**Note:** Panel A shows the mean exercise time per exercise session for all patients. Panel B shows the mean exercise time per day for the patients who stopped exercising before they reached 30 minutes of exercise per day (i.e., terminating exercise early). Data are in means with a 95% CI. Panel A: The booklet exercise group: day 1 (n = 63), day 2 (n = 43), day 3 (n = 34), day 4 (n = 25), day 5 (n = 18), day 6 (n = 14), day 7 (n = 11), and day 8 (n = 8). The in-bed cycling group: day 1 (n = 61), day 2 (n = 43), day 3 (n = 37), day 4 (n = 26), day 5 (n = 20), day 6 (n = 16), day 7 (n = 12), and day 8 (n = 11). Panel B: The booklet exercise group: day 1 (n = 49), day 2 (n = 30), day 3 (n = 21), day 4 (n = 17), day 5 (n = 14), day 6 (n = 10), day 7 (n = 8), and day 8 (n = 6). The in-bed cycling group: day 1 (n = 31), day 2 (n = 24), day 3 (n = 19), day 4 (n = 11), day 5 (n = 9), day 6 (n = 7), day 7 (n = 7), and day 8 (n = 8).

**Table S2.** Percentage decrease in length of stay and risk of 90-day readmission and 180-day mortality for patients with non-SARS-CoV-2 in the in-bed cycling, booklet exercise, and combined exercise training group compared to standard care.

|  | ***Model 1*** |  | ***Model 2*** |  | ***Model 3*** |  | ***Model 4*** |  |
| --- | --- | --- | --- | --- | --- | --- | --- | --- |
|  | Parameter estimates  (95% CI) | P-value | Parameter estimates  (95% CI) | P-value | Parameter estimates  (95% CI) | P-value | Parameter estimates  (95% CI) | P-value |
| ***Intention-to-treat analyses*** |  |  |  |  |  |  |  |  |
| *Length of stay* |  |  |  |  |  |  |  |  |
| Standard care, n=54 | Ref. |  | Ref. |  | Ref. |  | Ref. |  |
| In-bed cycling, n=52 | 0.91 (0.70–1.19) | 0.50 | 0.95 (0.73–1.23) | 0.68 | 0.95 (0.74–1.24) | 0.72 | 0.95 (0.73–1.24) | 0.72 |
| Booklet exercise, n=54 | 0.92 (0.71–1.21) | 0.56 | 0.95 (0.73–1.22) | 0.67 | 0.94 (0.73–1.22) | 0.66 | 0.93 (0.72–1.20) | 0.58 |
|  |  |  |  |  |  |  |  |  |
| Standard care, n=54 | Ref. |  | Ref. |  | Ref. |  | Ref. |  |
| Exercise, n=106 | 0.92 (0.73–1.16) | 0.47 | 0.95 (0.76–1.18) | 0.62 | 0.95 (0.76–1.19) | 0.64 | 0.94 (0.75–1.18) | 0.59 |
|  | ***Model 1*** |  | ***Model 2*** |  | ***Model 3*** |  | ***Model 4*** |  |
|  | Hazard ratio  (95% CI) | P-value | Hazard ratio  (95% CI) | P-value | Hazard ratio  (95% CI) | P-value | Hazard ratio  (95% CI) | P-value |
| ***Available-case analyses*** |  |  |  |  |  |  |  |  |
| *90-day readmission* |  |  |  |  |  |  |  |  |
| Standard care, n=51 | Ref. |  | Ref. |  | Ref. |  |  |  |
| In-bed cycling, n=51 | 0.71 (0.35–1.42) | 0.33 | 0.70 (0.35–1.41) | 0.32 | 0.68 (0.34–1.37) | 0.28 |  |  |
| Booklet exercise, n=52 | 0.61 (0.29–1.26) | 0.18 | 0.62 (0.30–1.29) | 0.20 | 0.62 (0.30–1.29) | 0.20 |  |  |
|  |  |  |  |  |  |  |  |  |
| Standard care, n=51 | Ref. |  | Ref. |  | Ref. |  |  |  |
| Exercise, n=103 | 0.66 (0.36–1.20) | 0.18 | 0.66 (0.36–1.21) | 0.18 | 0.65 (0.36–1.19) | 0.16 |  |  |
|  |  |  |  |  |  |  |  |  |
| *180-day mortality* |  |  |  |  |  |  |  |  |
| Standard care, n=51 | Ref. |  | Ref. |  | Ref. |  |  |  |
| In-bed cycling, n=51 | 0.65 (0.18–2.30) | 0.50 | 0.63 (0.18–2.25) | 0.48 | 0.59 (0.17–2.09) | 0.41 |  |  |
| Booklet exercise, n=52 | 1.00 (0.33–3.10) | 1.00 | 0.99 (0.32–3.08) | 0.99 | 0.92 (0.29–2.88) | 0.89 |  |  |
|  |  |  |  |  |  |  |  |  |
| Standard care, n=51 | Ref. |  | Ref. |  | Ref. |  |  |  |
| Exercise, n=103 | 0.82 (0.30–2.26) | 0.70 | 0.81 (0.29–2.23) | 0.68 | 0.75 (0.27–2.07) | 0.58 |  |  |

**Note:** The analysis did not include patients with CAP due to SARS-CoV-2. The outcome variable length of stay was logarithm-transformed before analysis, and the regression coefficients were back-transformed to provide ratios. Data were analysed with an analysis of covariance to quantify differences in length of stay between the standard care group and the in-bed cycling or booklet exercise group. The outcome variables readmission and mortality were analysed with Cox proportional hazard regression to assess differences in time to readmission and mortality between the standard care group and the in-bed cycling and booklet exercise groups. Additional analyses were made with a combined exercise training group, which is the in-bed cycling and booklet exercise groups combined as one group. Model adjustments according to the different models: *model 1*: unadjusted model; *model 2*: adjusted for age and sex; *model 3*: adjusted for age, sex, and number of comorbidities (0, 1, or ≥2); and *model 4*: adjusted for age, sex, CURB-65, BMI, and number of comorbidities (0, 1, or ≥2).

**Table S3.** Percentage decrease in length of stay and risk of 90-day readmission and 180-day mortality for patients who maintained an acceptable SpO_2_ during exercise in the in-bed cycling, booklet exercise, and combined exercise training group compared to standard care.

|  | ***Model 1*** |  | ***Model 2*** |  | ***Model 3*** |  | ***Model 4*** |  |
| --- | --- | --- | --- | --- | --- | --- | --- | --- |
|  | Parameter estimates  (95% CI) | P-value | Parameter estimates  (95% CI) | P-value | Parameter estimates  (95% CI) | P-value | Parameter estimates  (95% CI) | P-value |
| ***Intention-to-treat analyses*** |  |  |  |  |  |  |  |  |
| *Length of stay* |  |  |  |  |  |  |  |  |
| Standard care, n=62 | Ref. |  | Ref. |  | Ref. |  | Ref. |  |
| In-bed cycling, n=52 | 0.86 (0.66–1.12) | 0.26 | 0.85 (0.66–1.10) | 0.22 | 0.86 (0.67–1.11) | 0.25 | 0.87 (0.67–1.12) | 0.27 |
| Booklet exercise, n=58 | 0.96 (0.74–1.23) | 0.72 | 0.97 (0.76–1.24) | 0.80 | 0.97 (0.76–1.25) | 0.83 | 0.97 (0.75–1.24) | 0.78 |
|  |  |  |  |  |  |  |  |  |
| Standard care, n=62 | Ref. |  | Ref. |  | Ref. |  | Ref. |  |
| Exercise, n=110 | 0.91 (0.73–1.13) | 0.40 | 0.91 (0.74–1.13) | 0.40 | 0.92 (0.74–1.14) | 0.44 | 0.92 (0.74–1.14) | 0.44 |
|  | ***Model 1*** |  | ***Model 2*** |  | ***Model 3*** |  | ***Model 4*** |  |
|  | Hazard ratio  (95% CI) | P-value | Hazard ratio  (95% CI) | P-value | Hazard ratio  (95% CI) | P-value | Hazard ratio  (95% CI) | P-value |
| ***Available-case analyses*** |  |  |  |  |  |  |  |  |
| *90-day readmission* |  |  |  |  |  |  |  |  |
| Standard care, n=59 | Ref. |  | Ref. |  | Ref. |  |  |  |
| In-bed cycling, n=50 | 0.66 (0.33–1.32) | 0.24 | 0.61 (0.30–1.21) | 0.17 | 0.58 (0.29–1.17) | 0.13 |  |  |
| Booklet exercise, n=56 | 0.60 (0.30–1.19) | 0.14 | 0.61 (0.31–1.22) | 0.17 | 0.60 (0.30–1.19) | 0.14 |  |  |
|  |  |  |  |  |  |  |  |  |
| Standard care, n=59 | Ref. |  | Ref. |  | Ref. |  |  |  |
| Exercise, n=106 | 0.63 (0.35–1.11) | 0.11 | 0.61 (0.34–1.09) | 0.10 | 0.59 (0.33–1.05) | 0.07 |  |  |
|  |  |  |  |  |  |  |  |  |
| *180-day mortality* |  |  |  |  |  |  |  |  |
| Standard care, n=59 | Ref. |  | Ref. |  | Ref. |  |  |  |
| In-bed cycling, n=50 | 0.98 (0.30–3.21) | 0.98 | 0.89 (0.27–2.95) | 0.85 | 0.82 (0.25–2.70) | 0.74 |  |  |
| Booklet exercise, n=56 | 1.08 (0.35–3.34) | 0.90 | 1.04 (0.33–3.22) | 0.95 | 0.92 (0.30–2.88) | 0.89 |  |  |
|  |  |  |  |  |  |  |  |  |
| Standard care, n=59 | Ref. |  | Ref. |  | Ref. |  |  |  |
| Exercise, n=106 | 1.03 (0.38–2.79) | 0.95 | 0.97 (0.36–2.62) | 0.95 | 0.87 (0.32–2.36) | 0.79 |  |  |

**Note:** Patients with CAP who had at least one episode of desaturation in SpO_2_ during exercise were excluded from the analysis. The outcome variable length of stay was logarithm-transformed before analysis, and the regression coefficients were back-transformed to provide ratios. Data were analysed with an analysis of covariance to quantify differences in length of stay between the standard care group and the in-bed cycling or booklet exercise group. The outcome variables readmission and mortality were analysed with Cox proportional hazard regression to assess differences in time to readmission and mortality between the standard care group and the in-bed cycling and booklet exercise groups. Additional analyses were made with a combined exercise training group, which is the in-bed cycling and booklet exercise groups combined as one group. Model adjustments according to the different models: *model 1*: unadjusted model; *model 2*: adjusted for age and sex; *model 3*: adjusted for age, sex, and number of comorbidities (0, 1, or ≥2); and *model 4*: adjusted for age, sex, CURB-65, BMI, and number of comorbidities (0, 1, or ≥2).

**References**

1. Hoffmann TC, Glasziou PP, Boutron I, et al. Better reporting of interventions: template for intervention description and replication (TIDieR) checklist and guide. BMJ **2014**; 348:g1687.

2. Region Nordjylland. PRI - Fysioterapi til patienter som indlægges med pneumoni eller patienter der indlægges under observation for samme. 2023. Available at: https://pri.rn.dk/Sider/15603.aspx. Accessed 23 January 2024.

3. Borg GA. Psychophysical bases of perceived exertion. Med Sci Sports Exerc **1982**; 14:377–381.

4. Centers for Disease Control and Prevention. Perceived Exertion (Borg Rating of Perceived Exertion Scale) | Physical Activity | CDC. 2022. Available at: https://www.cdc.gov/physicalactivity/basics/measuring/exertion.htm. Accessed 26 January 2024.

5. Pedersen BK, Zacho M. Syg men sund og aktiv. 1st ed. Bianco Luno, Available at: https://www.academicbooks.dk/da/content/syg-men-sund-og-aktiv. Accessed 22 September 2023.

6. Mundy LM, Leet TL, Darst K, Schnitzler MA, Dunagan WC. Early mobilization of patients hospitalized with community-acquired pneumonia. Chest **2003**; 124:883–889.

7. Burtin C, Clerckx B, Robbeets C, et al. Early exercise in critically ill patients enhances short-term functional recovery. Crit Care Med **2009**; 37:2499–2505.
